# Distinctions in gastric cancer gene expression signatures derived from laser capture microdissection *versus *histologic macrodissection

**DOI:** 10.1186/1755-8794-4-48

**Published:** 2011-06-02

**Authors:** Hark Kyun Kim, Joseph Kim, Susie Korolevich, Il Ju Choi, Chang Hee Kim, David J Munroe, Jeffrey E Green

**Affiliations:** 1 National Cancer Institute, Bethesda, MD, 20892, USA; 2 National Cancer Center, Goyang, Gyeonggi, 410-769, Republic of Korea; 3 SAIC-Frederick, Inc., National Cancer Institute-Frederick, Frederick, MD, 21701, USA

## Abstract

**Background:**

Gastric cancer samples obtained by histologic macrodissection contain a relatively high stromal content that may significantly influence gene expression profiles. Differences between the gene expression signature derived from macrodissected gastric cancer samples and the signature obtained from isolated gastric cancer epithelial cells from the same biopsies using laser-capture microdissection (LCM) were evaluated for their potential experimental biases.

**Methods:**

RNA was isolated from frozen tissue samples of gastric cancer biopsies from 20 patients using both histologic macrodissection and LCM techniques. RNA from LCM was subject to an additional round of T7 RNA amplification. Expression profiling was performed using Affymetrix HG-U133A arrays. Genes identified in the expression signatures from each tissue processing method were compared to the set of genes contained within chromosomal regions found to harbor copy number aberrations in the tumor samples by array CGH and to proteins previously identified as being overexpressed in gastric cancer.

**Results:**

Genes shown to have increased copy number in gastric cancer were also found to be overexpressed in samples obtained by macrodissection (LS *P *value < 10^-5^), but not in array data generated using microdissection. A set of 58 previously identified genes overexpressed in gastric cancer was also enriched in the gene signature identified by macrodissection (LS *P *< 10^-5^), but not in the signature identified by microdissection (LS *P *= 0.013). In contrast, 66 genes previously reported to be underexpressed in gastric cancer were enriched in the gene signature identified by microdissection (LS *P *< 10^-5^), but not in the signature identified by macrodissection (LS *P *= 0.89).

**Conclusions:**

The tumor sampling technique biases the microarray results. LCM may be a more sensitive collection and processing method for the identification of potential tumor suppressor gene candidates in gastric cancer using expression profiling.

## Background

A major aim of microarray analysis is the identification of differentially expressed genes in subsets of clinical samples to match specific therapies to tumor subtypes. However, quantitative expression array analysis of clinical cancer samples with high stromal content is challenging since the ratio of epithelial tumor cells to stromal cells can vary greatly. Contaminating stroma may confound microarray-based expression and copy number analyses. Laser capture microdissection (LCM) is a valuable technique that enables one to isolate epithelial cells from stromal cells, thus enriching for epithelial content. The quantity of sample and RNA obtained by LCM is often quite limited, however, and requires an amplification step to generate sufficient material for microarray analyses. This amplification process may bias the results and lead to a skewed set of differentially expressed genes [1]. Histologic macrodissection (samples collected from tissue sections guided by microscopic analysis of a stained serial section) provides a larger amount of sample material compared to LCM that can obviate the need for an additional round of RNA amplification. However, macrodissected samples contain significantly more stromal cell content than samples obtained by microdissection.

Previous studies have compared these two tissue processing methods for clinical cancer samples. Based upon data from 14 rectal adenocarcinoma samples, Bruin *et al*. favored macrodissection over microdissection because of the relatively low contribution of stromal components in macrodissected samples from this tumor type and the biased gene expression results from microdissected samples due to the amplification of the RNA required for these samples [2]. On the other hand, Klee *et al*. suggested that microdissection profiling uniquely identify a large number of differentially expressed genes not otherwise found using bulk tissue sampling, based on data from 10 lung adenocarcinomas and 6 adjacent normal samples [3]. These studies were limited by small sample sizes, and, therefore, require further validation. It is also unclear whether the genes identified uniquely using microdissected samples represent useful biomarkers. Bias resulting from RNA amplification must be balanced against the benefit of enriching samples for epithelial content in considering whether microdissection is advantageous for expression profiling of tumors with high stromal content, such as gastric or pancreatic adenocarcinomas.

Microdissection is particularly useful for enriching gastric cancer tumor cells obtained from endoscopic biopsy samples, especially from diffuse-type gastric cancer which is composed of scattered tumor cells mixed with inflammatory cells and fibrosis. The decline in overall incidence of gastric carcinoma in U.S. during this century appears to be largely attributable to a decrease of the intestinal type lesions, while the occurrence of diffuse type is thought to have remained the same [4]. Using samples obtained by LCM, Wu *et al*. reported that malignant versus benign gastric epithelial cells could be distinguished with an accuracy of 99% based upon a 504 gene predictor [5]. This predictor included well-known genes expressed in the gastric epithelium including Trefoil factors 1, 2, and 3 [5]. Using LCM, Jinawath *et al*. identified 46 genes that may represent distinct molecular signatures for the two histological types of gastric cancer - diffuse-type and intestinal-type gastric cancers [6]. However, no studies have been performed to date directly comparing the macrodissection *vs*. LCM methods using the same set of gastric cancer samples.

In this study, we have sought to evaluate the distinctions between expression profiles derived from the same tumors that were processed by both macrodissection and LCM for microarray analyses. Given the difficulty in validating all of the differentially expressed genes identified using each type of sample collection, we compared the genes identified through our microarray analyses with proteins known to be overexpressed in gastric cancer. Additionally, we determined whether expression of genes identified in each signature correlated with alterations in the gene copy number that were identified by array comparative genomic hybridization (CGH) from the same tumors. Previous studies of gastric cancer have demonstrated a high correlation between array CGH and expression array data [7]. Copy number changes were evaluated using macrodissected tumor DNA in order to avoid the bias from whole genome amplification. We further determined whether the gene signatures we obtained from each sample collection and processing method were enriched for proteins that have previously been reported to be dysregulated genes in gastric cancer. Our results indicate that the LCM method is more sensitive for identifying genes that are underexpressed in the cancer compared to normal tissue (potential tumor suppressors), whereas macrodissection identifies more genes that are overexpressed in cancer. Therefore, macrodissection and LCM microdissection appear useful for studying different aspects of cancer biology.

## Methods

### Patients

Twenty patients who were analyzed in this study is a part of 96 patients who participated in a prospective study and whose samples were used as an expression training set to develop a chemo-response predictor [8]. Part of expression and CGH array data from their macrodissected samples was previously reported [8,9]. Sample collection, treatment, and follow-up were performed according a protocol approved by the Institutional Review Board (IRB) of the National Cancer Center Hospital in Goyang, Korea (NCCNHS01-003). All patients signed an IRB-approved informed consent form. Eligibility for enrollment into the study included the following parameters: 1) age ≥ 18 years; 2) histologically-confirmed gastric adenocarcinoma; 3) clinically-documented distant metastasis; 4) no previous or concomitant malignancies other than the gastric cancer; 5) no prior history of chemotherapy, either adjuvant or palliative; and 6) adequate function of all major organs. Patients received cisplatin 60 mg/m^2 ^IV on day 1 and fluorouracil 1,000 mg/m^2 ^IV on days 1-5 of a 3-week schedule.

### Tissue processing

Before macrodissection, tumor samples had median tumor nuclei of 50% (interquartile range, 30-60%). Macrodissection was performed as previously described [10]. Macrodissection lead to average 60% of tumor nuclei at top slide (interquartile range, 60-72.5%). For microdissection, tumor and normal tissue sample cryosections were cut at 10 μm, and stored frozen at -80°C. Slides were dehydrated using nuclease-free HistoGene (Molecular Devices, Sunnyvale, CA) reagents according to the manufacturer's recommendations. Microdissection was performed using PixCell II (Arcturus Bioscience, Mountain View, CA). Dehydration and LCM was limited to 15 min or less for each sample collected. A total of 10,000 laser shots (spot size of 15 μm in diameter) were collected using CapSure Macro LCM Caps for each sample. RNA was isolated using PicoPure RNA Isolation Kit (Molecular Devices). Briefly, the epithelial cells were incubated with 50 μL of extraction buffer in a 0.5 mL microcentrifuge tube at 42°C for 30 min. DNase (QIAGEN, Valencia, CA) treatment was performed directly within the purification column, and the RNA was isolated using the elution volume of 8 μL (Molecular Devices). Five μL of RNA from microdissected cell populations was converted to biotinylated, antisense cRNA target, using the Affymetrix two-cycle labeling method (Santa Clara, CA). All biotinylated targets were fragmented and 15*μ*g of each was hybridized to HG-U133A GeneChip microarrays following the manufacturer's protocol. Scanned array images were reviewed and converted to signal data using the Affymetrix MAS 5.0 algorithm.

### Array CGH

Genomic DNA was extracted from samples using TRI reagent (Invitrogen, Carlsbad, CA), according to the manufacturer's protocol, and additionally purified using the QIAamp DNA Micro Kit (QIAGEN). For array CGH experiments, Agilent 4x44k HD-CGH Microarrays containing 44,000 features (Agilent Technologies, Santa Clara, CA) were used. 0.5-1 μg of tumor genomic DNA samples and the same amount of human genomic DNA from multiple anonymous female donors (Promega, Madison, WI) were digested with AluI (50 units) and RsaI (50 units) for 2 h at 37°C. 5 μl of Random Primer was mixed with the digested DNA template. The reference and sample DNA were labeled using Agilent's Labeling Kit PLUS, which includes 5x buffer, 10x dNTP, Cy-3/5 dUTP (1.0 mM), and Exo-Klenow Fragment. The probe mixture of Cy3 labeled sample DNA, Cy5 labeled reference DNA (39 μl), 5 μl of human Cot-1 DNA (Invitrogen), 11 μl of Agilent 10 × blocking agent and 50 μl of Agilent 2 × hybridization buffer was denatured at 95 °C for 3 min and incubated at 37 °C for 30 min. The probe was applied to the array using an Agilent microarray hybridization chamber, and hybridized for 21 h at 65°C in a rotating oven at 20 rpm. Arrays were washed according to the manufacturer's recommendations, dipped in Agilent's stabilizing and drying solution, and scanned using an Agilent 2565AA DNA microarray scanner. The Agilent's Scan Program Control Program 7.0 and Agilent's Feature Extraction Software Program 9.5.1 were used for data processing. Array CGH data were analyzed using Agilent's CGH Analytics software (version 3.5.14). ADM-2 algorithm with threshold 6, with fuzzy zero and centralization on, was used to identify aberration. Criteria for aberration filtering were minimum probes of 5, minimum average absolute log_2 _ratio of 0.5, and maximum aberrations of 1,000,000. Aberrations identified for each sample were listed and graphically displayed.

### Gastric cancer genes in the literature

To generate a user-defined gene set for our gene comparison analyses, we searched PubMed database for genes with gastric cancer cell-specific protein expression, using keywords of "gastric cancer", "immunohistochemistry" and "overexpressed" or "loss of expression". For our gene set comparison analyses, gene symbols of gastric cancer specific genes were mapped to probe set IDs on the HG-U133A array (http://www.NetAffx.com). There were 178 ("overexpressed") and 327 ("loss of expression") articles in the Pubmed at the time of writing.

### Statistical analysis on expression array data

Affymetrix HG-U133A gene expression microarray data were analyzed with gene set comparison analysis algorithms of BRB ArrayTools (version 3.8, National Cancer Institute, http://linus.nci.nih.gov/BRB-ArrayTools.html) [11]. The gene set comparison tool analyzes user-defined gene sets for differential expression among pre-defined classes (*i.e*., cancer *vs*. normal) of a source dataset. User-defined gene sets used in this study include U133A probe sets corresponding to genes with copy number change and corresponding to gastric cancer genes in the literature. Genes whose tumor/normal log_2 _ratio is higher than 0.5 in at least one of 20 patient samples were included in the list of genes with copy number gain. Similarly, genes with copy number loss (log_2 _ratio < -0.5) were listed. These user-defined gene sets were analyzed for differential expression between 20 cancer samples and 6 normal samples (*i.e*., 3 macrodissected and 3 microdissected samples).

For each source dataset, a *P*-value is computed for each gene to correlate the expression level for the differential expression between pre-defined classes, generating a ranked gene list of a given BRB-ArrayTools project. For a set of *N *genes, the least squares (LS) statistic is defined as the mean negative natural logarithm of the *P*-values of the appropriate single gene univariate tests [12]. A summary statistic is computed that summarizes these *P *values over the user-defined gene set; the summary statistic is average log(*P*) for the LS summary of how the *P *values differ from a uniform distribution for LS [12]. The summary statistic is related to the distribution of the summary statistics for random samples of *N *genes, sampled from those represented on the array. Here *N *is the number of genes in the user-defined gene set. 100,000 random gene sets were sampled to compute this distribution. The LS *P *value is the proportion of random sets of *N *genes with smaller average summary statistics than the LS summaries computed for the real data.

BRB-ArrayTools estimated LS *P *values for enrichment of the 4 gene sets in our gastric cancer transcriptome signature identified by each tissue processing method as follows. First, in order to compare the 2,324 genes associated with copy number gain with our gastric cancer transcriptome signature identified by the microdissection, the LS statistic of 2,324 amplified genes was estimated by computing a mean negative natural logarithm of the *P *values of the single gene univariate tests for differential expression of each of 2,324 genes between 20 microdissected gastric cancer samples and 6 normal samples. Then BRB-ArrayTools calculated the proportion of random sets of 2,324 genes with smaller average summary statistics than the LS summaries computed for the real data (LS *P *value). The gastric cancer transcriptome signature identified by the microdissection was also compared with 677 genes associated with the copy number loss, 58 proteins reported to be overexpressed in gastric cancer, and 66 proteins reported to be underexpressed in gastric cancer, with respective LS *P *values. LS *P *value less than 0.01 was considered significant. The same analyses were repeated for gastric cancer transcriptome signature identified by the macrodissection method.

### Immunohistochemistry

TFF1 immunohistochemistry was performed using surgical or endoscopic biopsy tissue samples from 16 gastric cancer patients (16 cancer and 2 adjacent normal tissue samples), and 4 healthy volunteers, who were not included in this DNA microarray study. Grossly-normal gastric mucosa tissue samples were collected from the gastric antrum of healthy volunteers using a blind biopsy technique, with informed consent [9]. Paraffin-embedded formalin-fixed tissue slides (4 μm thick) were stained with 13734-1-AP (ProteinTech Group, Chicago, IL) at 1:50 for 60 min at room temperature and Envision anti-rabbit horseradish peroxidase **(**K4003, DAKO, Carpinteria, CA**) **for 30 min at RT. The reaction was visualized using diaminobenzidine (K3468, DAKO) and counterstained with hematoxylin. TFF1 expression was evaluated semi-quantitatively at 200*x *magnification, based on percentage of positively stained cells ("-" = immunostaining in ≤ 10% of cells; "+" = 11-50%; "++" = 51-75%; "+++" = 76-100%) [13,14]. Immunostaining without primary antibody and normal gastric epithelium of a control tissue microarray served as negative and positive controls, respectively [15]. Cytoplasmic stain which was unequivocally deeper than background was counted as positive.

## Results

### Determination of global gene expression signatures from macrodissected and LCM samples

**Figure 1:**
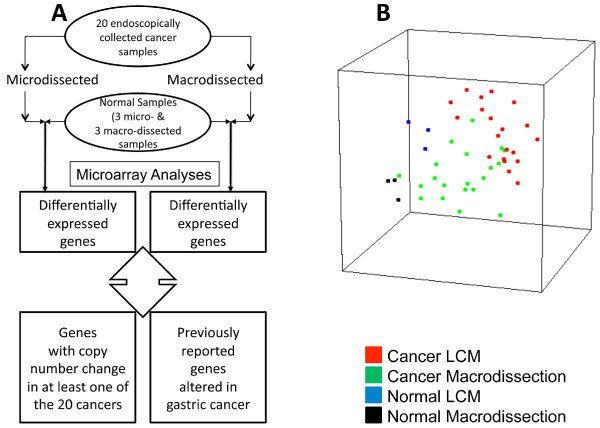
**(A) Study scheme of sample collection and microarray processing (B) Principal component analysis of gene expression profiles of micro- and macro-dissected tumor samples from 20 gastric cancer patients and 6 normal samples from healthy volunteers**.

Table 1 delineates the clinico-pathological characteristics of the patients and volunteers included in this microarray study. Microarray data was obtained for both LCM and macrodissected samples from the same 20 biopsies (Figure 1A). Although of acceptable quality, microarray data from LCM samples had generally lower "present call" than macrodissected samples (*data not shown*). Principal component analysis of the global gene expression patterns derived from the micro- and macro-dissected gastric cancers, and the normal samples demonstrated a clear separation of each sample group (Figure 1B). The median Pearson correlation between the two processing methods was 0.75 (interquartile range, 0.71-0.81).

**Table 1:** Clinico-pathological characteristics of patients and volunteers included in microarray analysis

	***Patients (n = 20)***	***Volunteers (n = 6)***
***Baseline clinico-pathological characteristics***		
Age-year		
Median	59	52
Interquartile range	54-69	43-61
Sex - no. (%)		
Male	16 (80%)	3 (50%)
Female	4 (20%)	3 (50%)
Performance status (PS) - no. (%)		
ECOG1 PS 0 or 1	20 (100%)	
Histological type - no. (%)		
Lauren's intestinal	6 (30%)	
Lauren's diffuse	14 (70%)	
Location of primary lesion - no. (%)		
Upper 1/3	4 (20%)	
Middle 1/3	6 (30%)	
Lower 1/3	10 (50%)	
Distant metastasis - no. (%)	20 (100%)	
***Treatment and outcome***		
Chemotherapy regimen - no. (%)		
Cisplatin/Fluorouracil	20 (100%)	
Overall survival - month.		
Median	8.0	
Interquartile range	5.6-14.7	
Time to progression - month.		
Median	3.5	
Interquartile range	2.3-6.2	

^1^Eastern Cooperative Oncology Group

Tables 2 and 3 show genes overexpressed in micro- and macro-dissected gastric cancer samples at feature selection *P *< 10^-6^. *Cell morphology *(*AIF1,E2F1, E2F3, KIR2DL1, KIRREL, NPR1, RUNX2, TRIO*) was the most enriched functional category of the 42 genes overexpressed in the microdissected samples compared to the normal samples (feature selection *P *< 10^-6^) as identified by Ingenuity Pathway Analysis (IPA) (Table 2). *Tumor morphology *(*APOE, BIRC5, CD14, COL1A1, COL1A2, CYR61, FKBP1A, IL8, MCAM, MIF, RHOB*) was the most enriched functional category among the 73 genes overexpressed in the macrodissected samples (feature selection *P *< 10^-6^) by IPA (Table 3). Extracellular matrix genes, such as *COL6A2, COL1A1, COL1A2*, and *COL5A2*, were prominent in the macrodissected samples and presumably contributed by the stromal cells. Table 4 shows genes underexpressed in micro- and macro-dissected gastric cancer samples at feature selection *P *< 10^-6^.

**Table 2:** Genes overexpressed in microdissected gastric cancer at feature selection *P *< 10^-6^

Gene	FC^1^	Gene	FC
BMP3	38.5^2^	HIST1H4C	6.3
BGN	30.3	LEPRE1	5.9
TRIO	18.5	ETNK2	5.6
GADD45GIP1	17.2	TRIP6	5.3
MIER2	16.7	FAM125B	5.3
KIFC3	16.4	NPR1	5.3
217318_x_at	13.7	DSCC1	5.3
217219_at	13.2	CLUL1	5.0
RUNX2	12.5	HMGB3	4.5
SMARCD1	12.0	E2F3	4.3
KIRREL	12.0	AIMP2	4.2
215621_s_at	12.0	ATAD5	4.0
GRM2	10.0	E2F1	3.8
FJX1	10.0	FKSG49	3.7
AIF1	10.0	DVL2	3.7
THY1	9.1	TIPRL	2.9
CARD10	9.1	EIF2C3	2.9
SIM2	9.1	NAT10	2.8
AIF1	9.1	MED27	2.7
APOBEC3G	8.3	PIN4	2.7
RHAG	8.3	CTPS	2.6

^1^fold change, defined by the expression ratio of cancer to normal (= cancer/normal)

^2^All these genes had false discovery rate <0.001.

**Table 3:** Genes overexpressed in macrodissected gastric cancer at feature selection *P *< 10^-6^

Gene	FC^1^	Gene	FC
LY6E	24.4^2^	SRM	6.7
IL8	22.7	NGLY1	6.7
CA12	20.0	RHOB	6.3
SBNO2	19.2	ACTN1	5.9
UBE2S	17.2	LOXL2	5.9
CYR61	17.2	COL5A2	5.9
ANGPT2	15.9	TRIM28	5.6
COL6A2	14.3	218982_s_at	5.6
BOP1	13.2	C7orf44	5.3
COL1A1	13.2	UBE2C	5.3
LPL	13.0	CEP76	5.3
MFGE8	12.8	BIRC5	5.3
APOE	12.2	PNO1	5.0
G6PC3	10.9	FSTL1	5.0
215900_at	10.3	GRINA	4.8
NUP62	10.0	MRTO4	4.8
MRPL4	10.0	STC1	4.8
GNL3L	10.0	MRPL12	4.5
MCAM	9.1	FKBP1A	4.5
PDLIM7	9.1	IFI30	4.5
216472_at	9.1	KPNA6	4.3
ACTN1	9.1	216532_x_at	4.3
BYSL	9.1	CENPI	4.2
GNAI2	8.3	PPM1G	4.2
NCAPH2	8.3	ICT1	3.7
CD14	8.3	SFRS14	3.6
EXOSC4	8.3	CTPS	3.6
OBFC2B	8.3	IMP4	3.3
PPP1R15A	7.7	UBE2G2	3.2
COL1A2	7.7	ISG20L2	3.2
GPX1	7.7	EIF4A1	3.1
MIF	7.7	HDGF	2.6
NME1	7.1	PSMD14 2.6	
PPIL2	7.1	220856_x_at	2.4
CCDC85B	7.1	CNOT3	2.4
SPARC	6.7	GLT25D1	2.0
C8orf55	6.7		

^1^fold change, defined by the expression ratio of cancer to normal (= cancer/normal)

^2^All these genes had false discovery rate <0.001.

**Table 4:** Genes underexpressed in micro- and macro-dissected gastric cancer at feature selection *P *< 10^-6^

**Microdissected**		**Macrodissected**	
**Gene**	**FC^1^**	**Gene**	**FC**
HPGD	-25.0^2^	208498_s_at	-11.1
HRASLS2	-20.0	SIDT2	-7.7
ABCC3	-20.0	MUC5AC	-5.9
SLC25A37	-16.7	CTAGE5	-5.0
ABHD2	-14.3	GNA11	-3.8
VIPR1	-10.0	ARFIP1	-3.4
CYTIP	-9.1	214316_x_at	-3.3
GALNT6	-9.1	222149_x_at	-3.3
SULT1A2	-9.1		
OAS1	-8.3		
PDCD4	-7.1		
NR3C2	-7.1		
DOCK6	-6.3		
SULT1A1	-5.9		
ZFYVE26	-5.9		
213212_x_at	-5.6		
DSCR3	-5.3		
TMEM131	-5.3		
ECHDC2	-5.0		
DENND1B	-5.0		
KIAA0141	-4.8		
RNF103	-4.8		
PDCD4	-4.5		
CABIN1	-4.5		
222371_at	-4.3		
RRBP1	-4.0		
CC2D1A	-3.8		
216438_s_at	-3.8		
SGSM3	-3.8		
ARPC2	-3.7		
TRAK1	-3.6		
GNA11	-3.6		
PAFAH1B1	-3.4		
CNDP2	-3.2		
SPOP	-3.1		
PARP4	-3.1		
ERLIN1	-2.9		

^1^fold change, defined by the negative of the expression ratio of normal to cancer (= - (normal/cancer))

^2^All these genes had false discovery rate <0.001.

### Comparison between expression and array CGH data

**Figure 2:**
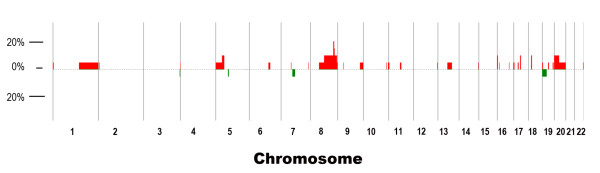
**Graphic image illustrating the percent frequency of probes detected (aberrations) among all 20 samples**.

Array CGH analysis was performed using genomic DNA extracted from macrodissected samples containing > 50% tumor cells (criteria used by previous studies [16,17]) since sufficient DNA could be obtained without the need for whole genome amplification as required for microdissected samples. Whole genome DNA amplification may potentially introduce artifactual bias in array CGH results [18]. Depicted in Figure 2 is the frequency of DNA copy number aberrations among all of the 20 samples. Our copy number aberration data was generally consistent with previously reported data [7,16,17,19-23]. Four of 20 patients had amplification of chr8 q24.13-q24.21 (126357475-128822596) which contains the *MYC *oncogene. The second most common amplification locus was chr17 q21.2 (36109939-36230163) which was amplified in 3 patients. Seven patients had no detectable chromosome aberrations. These 7 samples contained a median of 70% tumor cells, while the other 13 patients had a median 60% tumor cells (*P *value = 0.1). Hence, the lack of detectable chromosome aberrations in the 7 samples was not due to a lower percentage of tumor cells in those samples.

**Figure 3:**
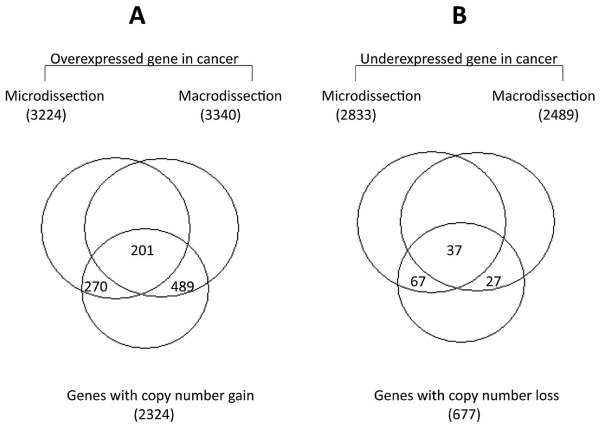
**Number of genes overlapping between LCM and macrodissected expression array datasets and array CGH data from the same set of 20 patients**.

There were 2,324 unique genes which were associated with copy number gain in at least one of the 20 patients, and 677 genes associated with copy number loss. Using gene set comparison analyses, we compared these gene sets with our transcriptome signatures identified by the different sample isolation methods. We hypothesized that dysregulated genes associated with copy number aberrations are more likely to be involved as contributors to oncogenesis rather than simply as "bystanders." Hence, the 2,324 genes contained within regions of copy number gains were analyzed with regard to their expression from array data obtained using the macrodissection method (feature selection *P *< 0.05). The overlap between the list of genes in regions of amplification and enrichment for their expression in samples that were macrodissected was statistically significant (LS *P *value = 10^-5^; see Methods for statistical description). However, this association was not observed when expression data was analyzed from microdissected samples (feature selection *P *< 0.05; LS *P *value = 0.41) (Figure 3A). Thus, there was stronger association between the pattern of copy number gain and gene expression only in samples that were macrodissected. For example, *MYC*, the most frequently amplified gene in our patient samples, was determined to be significantly overexpressed in macrodissected samples, but not in those that were microdissected, although this result may be due to relatively small sample size or heterogeneity within the tumor.

A list of 677 genes was identified in regions where DNA copy number loss was found in at least one of the 20 study patients. The expression of these genes was analyzed in the macro- and micro-dissected array datasets. A significant association was found between the genes with loss of copy number and their expression in both macro- and micro-dissected samples. (LS *P *values, 0.009 and 0.006 for LCM and macrodissected samples, respectively) (Figure 3B).

### Concordance of gene signatures with genes previously reported to be associated with gastric cancer

A PubMed literature search was performed to identify previously reported over- and under-expressed genes and proteins for gastric cancer (keywords: immunohistochemistry, gastric cancer, and overexpressed and or loss of expression). 58 proteins overexpressed in gastric cancer were identified in this manner. Gene expression for these 58 proteins were found to be enriched in expression data from samples collected by macrodissection (LS *P *< 10^-5^), but no enrichment in expression of the 58 genes was found for samples collected by microdissection (LS *P *= 0.013). In contrast, 66 proteins reported to be underexpressed in gastric cancer were enriched in expression array data from samples collected by microdissection (LS *P *< 10^-5^), but not from samples collected by macrodissection (LS *P *= 0.89) (Table 5).

**Table 5:** Gastric cancer genes in the literature that were differentially expressed between 20 cancer and 6 normal samples at feature selection *P *< 0.05 according to microarray data generated using each tissue processing method

Overexpressed genes in cancer			Underexpressed genes in cancer		
LCM&Macro^1^	LCM	Macro	LCM&Macro	LCM^2^	Macro
APOE	EGFR	AKT1	ANXA10	ANXA7	CDKN2B
AURKA	HGF	ANXA2	CASP6	BAD	FHIT
CCNE1	MET	CALR	CASP7	HLA-B	
CDC20	RHOA	CCNB1	CDH1	HLA-E	
CDC25B	TNS4	EEF2	CTNNA1	HLA-G	
CXCR4		ESM1	GSN	PRSS8	
E2F1		HIF1A	HLA-F	PTEN	
EGR1		MINA	IQGAP2	SDHB	
GRB2		PHB	KCNE2	SH3GLB1	
HK2			KLF4	TFF1	
ICAM1			MUC6		
INHBA			RARB		
LOXL2			SMAD4		
MCM3					
PTMA					
SPARC					

^1^Previously reported overexpressed proteins for gastric cancer which were also overexpressed in our microdissected (LCM) and macrodissected cancer samples as compared with normal samples

^2^Previoulsy reported underexpressed proteins for gastric cancer which were also underexpressed in our microdissected (LCM) cancer samples, but not in macrodissected cancer samples

### Validation of microarray data

**Figure 4:**
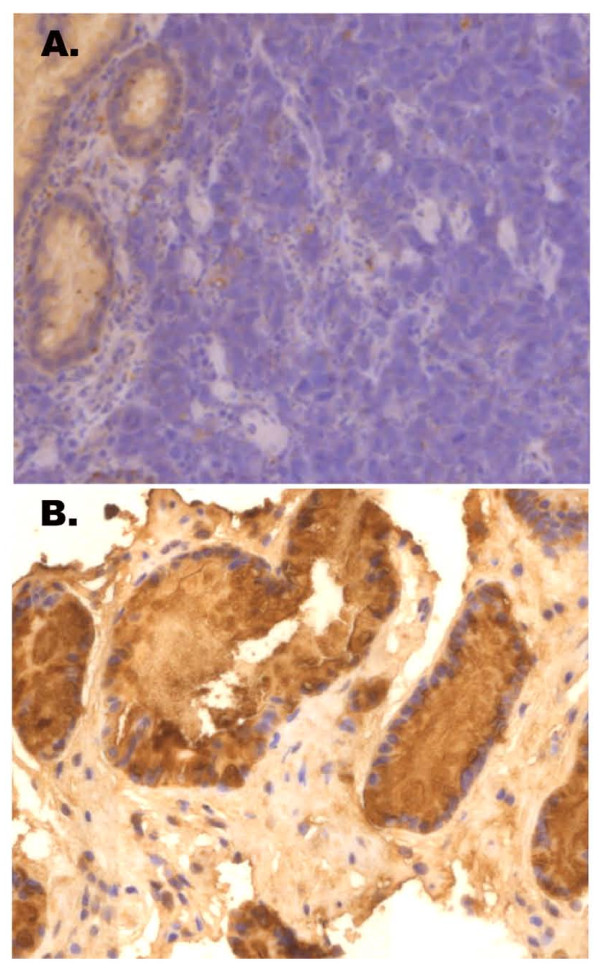
**Representative TFF1 immunohistochemical staining results for (A) a gastric adenocarcinoma demonstrating the loss of TFF1 expression, and (B) normal gastric mucosa from a healthy volunteer expressing TFF1 in gastric epithelial cells**. (Magnification = 200x)

In order to validate our microarray data, we performed immunohistochemical analyses on a gene identified as underexpressed in microdissected cancer samples from 16 patients and 4 volunteers who were not included in DNA microarray study. *TFF1 *was chosen for this immunohistochemistry validation study, because it is significantly underexpressed in the LCM samples (*P *= 0.0036) but not in macrodissected samples (*P *= 0.09), as compared with normal gastric mucosa. TFF1 immunoreactivity in cancer was evaluated as -, +, ++, and +++ in 7 (43.8%), 3 (18.7%), 4 (25.0%), and 2 (12.5%), respectively. In contrast, all of the 6 normal gastric mucosa samples (4 healthy volunteers and 2 normal adjacent tissue samples) preserved TFF1 immunoreactivity (+++) (Figure 4). Thus, gastric cancer samples had significantly lower TFF1 immunoreactivity than normal gastric mucosa, consistent with previous reports [13,14] (*P *for chi-square = 0.007).

These results demonstrate that macrodissection-based gene expression analyses outperformed gene expression analyses from LCM samples for identifying genes overexpressed in gastric cancer. In contrast, microdissection outperformed macrodissection for the identification of possible gastric cancer tumor suppressor genes.

## Discussion

Although limited by a relatively small sample size, this study demonstrates the feasibility of LCM as a tissue collection and processing method for gastric cancer samples with high stromal content. Gene signatures identified from microdissected samples were moderately correlated with signatures identified from macrodissected samples from the same biopsy samples. Most genes previously found to be associated with gastric cancer were identified in the signatures from both sets of samples (LS *P *value < 0.001 for both sets). Importantly, microdissection was found to be a better tissue processing technique than macrodissection for identifying down-regulated genes including potential tumor suppressor genes in gastric cancer.

Interestingly, samples obtained by macrodissection were enriched for genes that were overexpressed, including potential oncogenes, compared to samples obtained by microdissection. This may be due, in part, to the inherent biases introduced by RNA amplification for LCM samples. The relative disadvantage of microdissection in identifying genes overexpressed in cancer may be related to the fact that an additional round of RNA amplification, required for processing microdissected samples, is associated with 30% transcript loss [1]. It is also possible that macrodissected samples contain a larger amount of tumor sample and, thus, capture a greater degree of molecular heterogeneity of the tumor compared to LCM samples. Thus, it would be more likely to include regions with increased gene expression in macrodissected samples compared to LCM samples. Additionally, macrodissected samples may contain normal stomach epithelium that expresses sufficiently large amounts of a gene as to mask the loss of gene expression in the tumor cells within the sample. Further, macrodissected samples include a significant portion of stromal cells that would express many additional genes not expressed in the epithelial components of the tumor. Our data, however, needs to be interpreted with some caution. Most of the genes within amplicons or previously identified as being overexpressed in gastric cancer exhibited a prominent (>2-fold) up-regulation by microarray in the cancer samples compared to normal controls. Since the macrodissected samples contained > 50% tumor cells, the most prominent changes in gene expression are likely identified using the macrodissected samples. However, more subtle increases in gene expression could have been missed.

In addition to providing more robust information about highly up-regulated genes in cancer cells, macrodissection has the advantage of providing important information from stoma. It has become clear that both the epithelial and stromal components of a tumor interact in important ways to determine the biologic properties of the cancer. Accumulating evidence suggests that alterations in the expression of genes in the stroma provide important prognostic information [24,25]. However, the limitation in sample size of this study did not allow us to compare the potential prognostic values of the signatures we identified using the two tissue processing methods.

On the other hand, samples obtained by microdissection were enriched for genes whose expression was reduced in the tumor samples compared to normal gastric epithelial cells. The downregulated genes we identified are in keeping with genes whose expressions have been reported to be reduced in gastric cancer. Therefore, microdissected samples appear better suited for identifying genes whose expression is reduced in gastric cancer, some of which may be potential tumor suppressor genes. This finding is consistent with the previous observation of Klee *et al*. that substantially more downregulated genes were uniquely identified in the LCM dataset than identified in the bulk dataset [3]. These authors suggested that this is reflective of the cell population sampled rather than amplification bias since this effect was not observed in the array data generated using linearly amplified bulk tumor RNA. Whether those genes that were uniquely identified in the LCM datasets are truly biologically significant genes or an artifact arising from the LCM process was not fully evaluated by DNA- or protein-based studies [3]. Tomlins *et al*. also reported that LCM cell sampling in prostate cancer minimized the strong contaminating influence of stromal component and positively effected the selection of down-regulated genes [26]. Our data confirms and extends previous observations by providing further evidence that downregulated genes identified by using microdissected material are likely to represent transcriptional changes specific to the epithelial tumor compartment and enriched for potential tumor suppressor genes, in keeping with the associated loss of DNA copy number as observed by array CGH.

Thus, we suggest that macrodissection and microdissection may be useful for evaluating different aspects of gastric cancer biology using DNA microarray. Although we could not validate our microarray data using different RNA methods such as the quantitative reverse transcription polymerase chain reaction (RT-PCR), our conclusion is supported by consistent results from multiple gene set comparison analyses. Very small amounts of sample could be obtained by endoscopic sampling approved by the IRB, which greatly limited the amount of RNA that was available for analysis. Therefore, we were unable to perform RT-PCR experiments that would otherwise have been done. Microarray data using the Affymetrix platform has been generally well correlated to RT-PCR data in many studies [27-29]. The fact that there was significant overlap between genes in our signatures and the gene lists we used for comparison provides indirect validation for our discriminatory genes.

## Conclusions

The tumor sampling technique biases the microarray results. Macrodissected samples provide a global picture of the tumor transcriptome including contributions from the stromal component which may be critical for understanding tumor biology, whereas samples collected by microdissection eliminate the stromal contribution and are particularly useful for identifying genes whose expression is reduced during tumorigenesis.

## Competing interests

The authors declare that they have no competing interests.

## Authors' contributions

HKK designed experiments and performed tissue dissections, RNA preparation, analyzed the microarray data and drafted manuscript; JK performed LCM; SK performed the array CGH experiments; IJC biopsied patients to obtain samples; CHK and DJM performed gene expression microarray experiments; JEG assisted with analyses and contributed to the writing of the manuscript. All authors read and approved the final manuscript.

## Pre-publication history

The pre-publication history for this paper can be accessed here:

http://www.biomedcentral.com/1755-8794/4/48/prepub
